# Impact of low skeletal muscle mass index and perioperative blood transfusion on the prognosis for HCC following curative resection

**DOI:** 10.1186/s12876-020-01472-z

**Published:** 2020-10-07

**Authors:** Tomoaki Bekki, Tomoyuki Abe, Hironobu Amano, Minoru Hattori, Tsuyoshi Kobayashi, Masahiro Nakahara, Hideki Ohdan, Toshio Noriyuki

**Affiliations:** 1 Department of Surgery, Onomichi General Hospital, 1-10-23 Hirahara, Onomichi, Hiroshima, Japan; 2 Department of Gastroenterological and Transplant Surgery, Graduate School of Biomedical and Health Sciences, Hiroshima University, Kasumi 1-2-3 Minami-ku, Hiroshima, Japan; 3 Advanced Medical Skills Training Center, Institute of Biomedical and Health Sciences, Hiroshima University, Hiroshima, Japan

**Keywords:** Blood transfusion, Hepatocellular carcinoma, Low SMI

## Abstract

**Background:**

This study aimed to assess the prognostic factors including low skeletal muscle mass index (SMI) and perioperative blood transfusion for patients with hepatocellular carcinoma (HCC) following curative surgery.

**Methods:**

This study included 139 patients with HCC who underwent hepatectomy between 2005 and 2016. Univariate and multivariate analyses were performed to identify variables associated with overall survival (OS) and recurrence-free survival (RFS).

**Results:**

Low SMI was significantly related with poor OS, while blood transfusion had a strong impact on RFS. The male ratio and body mass index in the low SMI group were significantly higher than those in the high SMI group. There were no significant differences in age, virus etiology, laboratory data, liver function, tumor makers, and operative variables between the groups. Tumor factors such as tumor diameter, tumor number, poor differentiation, and intrahepatic metastasis (IM) did not significantly differ between the two groups. Operation time, intraoperative blood loss volume, and recurrence ratio were significantly higher in the blood transfusion group than in the non-transfusion group. IM was associated with poor OS and RFS.

**Conclusions:**

Low SMI and blood transfusion were independently related with long-term prognosis in patients with HCC following curative surgery.

## Background

Hepatocellular carcinoma (HCC) is the sixth most common carcinoma and third leading cause of cancer-related deaths worldwide [1]. Progression of surgical techniques and a better understanding of liver anatomy have played an important role in suppressing intraoperative blood loss [2, 3]. However, there is still substantial risk of perioperative blood loss in patients who undergo major hepatectomy, and the need for blood transfusion remains high [2]. From the aspect of immune surveillance for cancer, we formulated the following two hypotheses why perioperative blood transfusion should be avoided: 1) allogenic blood transfusion can increase the risk of virus infection, such as hepatitis B (HBV), hepatitis C, and human immunodeficiency syndrome [4, 5] and 2) it increases the risk of immunological complications due to postoperative infection, possibly leading to reduced long-term survival. Several studies have revealed that perioperative blood transfusions decreased the recurrence-free survival (RFS) and overall survival (OS) of patients after hepatectomy [6, 7]. Other reports have shown that perioperative blood transfusions does not influence RFS, OS, and disease-free survival (DFS) after hepatectomy [8, 9]. The influence of perioperative blood transfusion on tumor recurrence remains controversial.

Well-known prognostic variables such as tumor marker, advanced tumor stage, and vascular invasion were evaluated. Recently, tumor-associated variables and liver function have been strongly related to long-term prognosis. Honmyo et al. [10] reported that the albumin–bilirubin grade and albumin–indocyanine green evaluation grade were not only independent prognostic factors but also associated with postoperative complications. Preoperative nutritional status and immunological status were associated with postoperative complications and outcomes of patients with HCC such as obesity, Glasgow Prognostic Score (GPS) score, and neutrophil-to-lymphocyte ratio (NLR) [11–14]. Sarcopenia in HCC is also a well-known factor affecting long-term prognosis, based on the age, deteriorated immune status, and tumor-bearing condition [15–17].

This retrospective study aimed to clarify the postoperative prognostic factors, especially blood transfusion and low skeletal muscle mass index (SMI), for HCC patients with Child–Pugh grade a following curative surgery.

## Methods

### Patients

**Fig. 1:**
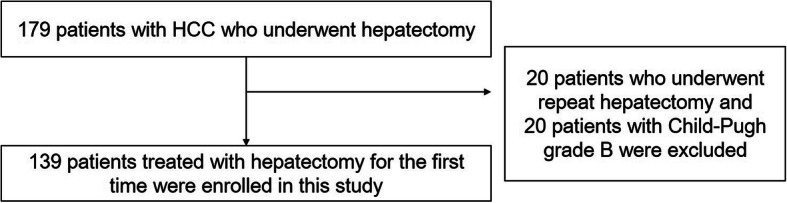
Study flowchart

Between 2005 and 2016, of the 175 patients with HCC who underwent hepatectomy at our institute, 139 patients who underwent hepatectomy for the first time were enrolled in this study. Patients with Child–Pugh grade B and who underwent repeat hepatectomy were excluded (Fig. 1). Following the guidelines of the Declaration of Helsinki (Fortaleza, Brazil, October 2013), this study was approved by the institutional review board of the Onomichi General Hospital (approval number: OJH201905).

### Perioperative blood transfusion

Perioperative blood transfusion was defined as transfusion of red blood cells (RBCs). This study did not involve the use of other blood products such as fresh frozen plasma and platelet concentrates during the perioperative period. Perioperative blood transfusion was defined as the use of RBCs within the period of patients’ hospitalization.

### Definition of low SMI

SMI was measured on an axial section at the third lumber vertebra (L3), which was taken 8 weeks prior to the surgery. They were segmented using standard Hounsfield unit (HU) ranges. Skeletal muscle was measured within the range of − 29 to + 150 HU, subcutaneous adipose was measured within the range of − 190 to − 30 HU, and abdominal adipose was measured within the range of − 150 to − 50 HU. Low SMI was defined as SMI < 52.4 cm^2^/m^2^ for men and < 38.5 cm^2^/m^2^ for women.

### Definition of intrahepatic metastasis and tumor number (solitary or multiple)

Intrahepatic metastasis (IM) was defined as a tumor derived from the primary tumor and located in the same segment with the primary tumor as multiple small satellite nodules [18]. Multiple tumors were defined as the other tumor which is different from the primary tumor.

### Treatment and follow-up

A follow-up blood examination to identify tumor markers was performed every 3 months after surgery for 5 years. Enhanced abdominal computed tomography (CT) was performed to rule out recurrence for 6 months. When HCC recurrence was suspected, magnetic resonance imaging was performed.

### Statistical analysis

Values for continuous variables were presented as median and range. Nominal variables were expressed as numbers (%). Non-parametric quantitative data were analysed using Mann–Whitney *U*-test. Chi-square test was performed to determine the relationship among nominal variables. The Kaplan-Meier method was used to analyze OS and DFS, and the log-rank test was used to compare different groups. Multivariate analyses were used to assess the factors that influenced OS and DFS by using the Cox regression model. *P*-values < 0.05 were considered significant.

To overcome bias owing to the different distribution of covariates among patients from the transfusion and non-transfusion groups, propensity score-matched analysis was performed using a multiple logistic regression model to predict the probability of each patient being transfusion on the basis of clinicopathological variables. Propensity scores were calculated according to baseline characteristics such as prognostic nutritional index (PNI), Hb, protein induced by vitamin K absence or antagonist-II (PIVKA-II), operation time, which were variables that differed significantly (*P*-values of < 0.05), based on logistic regression models for comparison with transfusion.

A 1:1 propensity score-match analysis was performed using the nearest-neighbor method to identify the impact of blood transfusion. Caliper was not set due to the small sample size. After 1:1 propensity score matching, to evaluate the discrimination and calibration abilities of propensity scores, C statistics and coefficient of determination of multiple logistic regression models (Nagalkerke R^2^) and the Hosmer–Lemeshow test were used. Model of Propensity score matching of transfusion had a good coefficient of determination (Nagalkerke *R*^2^ =  0.292), and was well calibrated (Hosmer–Lemeshow test; *p* = 0.798) with good discrimination [C statistic = 0.821, 95% confidence interval (CI) 0.726–0.915, *p* < 0.001]. This One-to-one pair matching was successful (0.523, 95% CI 0.340–0.705; *p* = 0.808). Calculations were performed using the SPSS software (version 24; IBM Corp., Armonk, NY, USA).

## Results

### Prognostic factors for OS and RFS identified by univariate and multivariate analyses

**Fig. 2:**
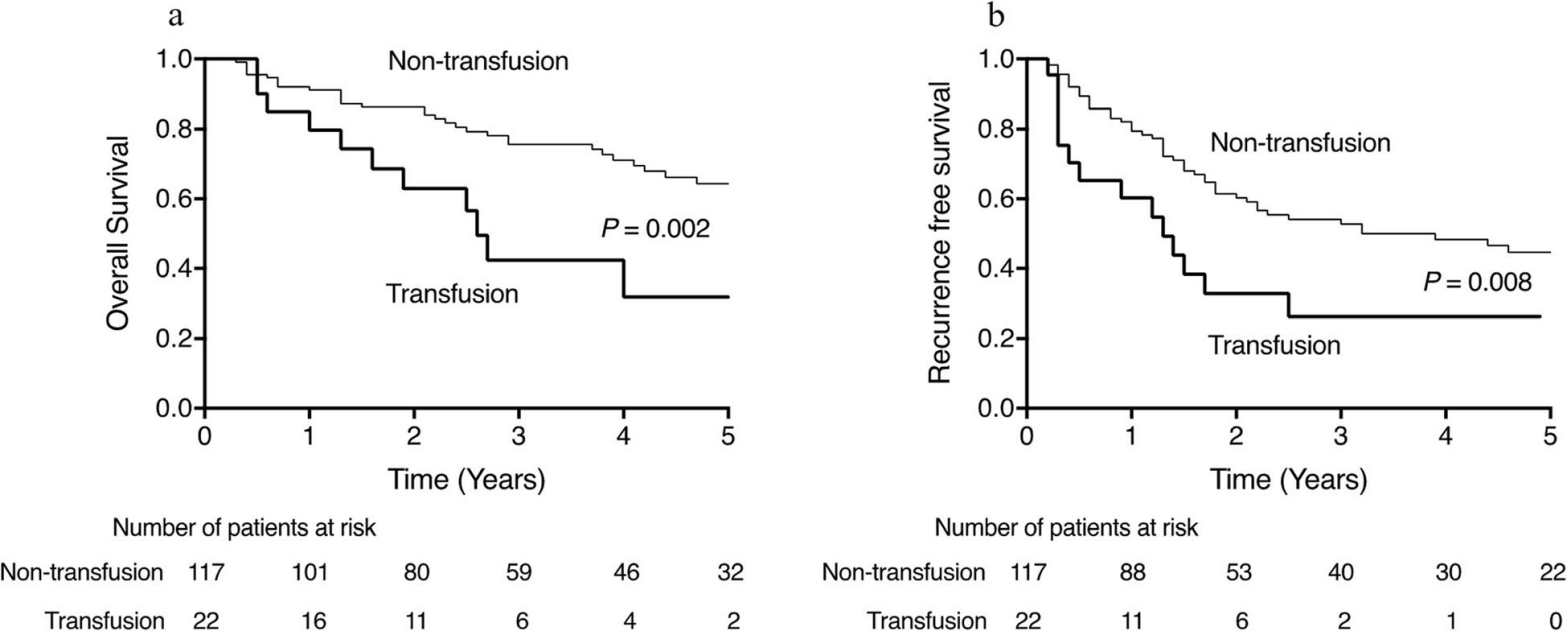
**a**, **b** Kaplan-Meier curve used to compare the transfusion group and non-transfusion group. The transfusion group had longer RFS than the non-transfusion group

The median postoperative follow-up duration was 2.7 years. The actual 1, 3, and 5-year OS rates were 90.3, 70.0, and 59.1%, respectively. The actual 1, 3, and 5-year RFS rates were 77.2, 47.9, and 39.7%, respectively. Table 1 presents the prognostic factors for OS. On univariate analysis, the following seven factors were statistically associated with poor OS: age > 80 years (*P* = 0.002), HBV (*P* = 0.027), elevated protein induced by vitamin K absence or antagonist (PIVKA-II) (*P* = 0.009), IM (*P* < 0.001), low SMI (*P* = 0.039; Supplemental Fig. 1a), blood transfusion (*P* = 0.002; Fig. 2a), and portal vein invasion (Vp) (*P* = 0.009). On multivariate analysis, the following four factors were revealed as independent poor prognostic factors of OS: age > 80 years (HR = 1.979; *P* = 0.035), HBV (HR = 1.681; *P* = 0.035), IM (HR = 3.675; *P* < 0.001) and low SMI (HR = 2.006; *P* = 0.046). Table 2 presents the prognostic factors for RFS. On univariate analysis, the following six variables were associated with poor RFS: elevated PIVKA-II (*P* = 0,048), elevated α-fetoprotein (AFP) (*P* = 0.036), tumor number (*P* = 0.025), IM (*P* < 0.001), Vp (*P* = 0.016), and blood transfusion (*P* = 0.008; Fig. 2b). On multivariate analysis, the following three factors were revealed as the poor prognostic factors of RFS: tumor number (HR = 1.810; *P* = 0.041), IM (HR = 4.115; *P* < 0.001) and blood transfusion (HR = 2.288; *P* = 0.008). There was no significant difference in RFS between patients with low SMI and those with high SMI. (*P* = 0.335; Supplemental Fig. 1b).

**Table 1:** Prognostic factors for overall survival identified by univariate and multivariate analyses (*n* = 139)

				Univariate	Multivariate		
Variables	n (%)	3-year survival	5-year survival	*P*-value	HR	95% CI	*P*-value
Male sex	110 (79%)	65.4%	56.7%	0.258			
Female sex	29 (21%)	88.5%	68.5%				
**Age (years)**							
**≤ 80**	109 (78%)	74.1%	66.8%	**0.002**	1.979	1.050–3.732	**0.035**
**> 80**	30 (22%)	54.1%	33.7%				
BMI (kg/m^2^)							
> 25	34 (25%)	74.4%	63.0%	0.441			
≤ 25	105 (75%)	68.7%	58.1%				
ALBI							
grade III	30 (22%)	64.2%	55.0%	0.677			
< grade III	109 (78%)	71.4%	59.6%				
**HBV**							
**(+)**	27 (19%)	78.3%	78.3%	**0.027**	1.681	1.037–2.726	**0.035**
**(−)**	112 (81%)	67.9%	52.7%				
HCV							
(+)	70 (50%)	71.5%	59.4%	0.668			
(−)	69 (50%)	68.0%	58.8%				
DM							
(+)	29 (21%)	75.3%	58.6%	0.582			
(−)	110 (79%)	68.6%	58.6%				
NLR							
≥ 4	13 (9%)	52.6%	52.6%	0.257			
< 4	126 (91%)	71.3%	60.0%				
PLT (×10^4^/μL)							
normal (13–35)	97 (70%)	73.4%	60.7%	0.582			
abnormal	42 (30%)	63.1%	55.4%				
Hb (g/dL)							
normal (13.5–15.8)	95 (68%)	74.2%	63.7%	0.094			
abnormal	44 (32%)	61.2%	50.1%				
PT (%)							
normal (70–130)	125 (90%)	72.3%	60.4%	0.172			
abnormal	14 (10%)	46.2%	46.2%				
AST (U/L)							
normal (13–33)	71 (51%)	78.6%	59.9%	0.086			
abnormal	68 (49%)	61.1%	56.5%				
ALT (U/L)							
normal (8–42)	100 (72%)	73.2%	59.7%	0.298			
abnormal	39 (28%)	61.8%	57.0%				
CHE (g/dL)							
normal (229–521)	77 (55%)	74.6%	64.0%	0.110			
abnormal	62 (45%)	63.6%	52.6%				
Alb (g/dL)							
normal (4.0–5.0)	115 (83%)	72.2%	60.3%	0.165			
abnormal	24 (17%)	58.4%	51.9%				
T-chol (mg/dL)							
normal (128–219)	118 (85%)	71.7%	59.1%	0.732			
abnormal	20 (14%)	66.9%	66.9%				
PIVKA-II (mAU/mL)							
normal (< 40)	62 (45%)	81.0%	74.7%	**0.009**	1.852	0.940–3.649	0.075
abnormal	77 (55%)	60.8%	45.7%				
AFP (ng/mL)							
normal (> 10)	68 (49%)	73.8%	66.9%	0.126			
abnormal	68 (49%)	66.2%	51.4%				
Tumor number							
solitary	113 (81%)	70.6%	65.5%	0.167			
multiple	26 (19%)	67.9%	37.0%				
Tumor size							
3 cm <	71 (51%)	68.0%	59.5%	0.185			
≤ 3 cm	54 (39%)	78.5%	71.5%				
Poor differentiation	20 (14%)	73.7%	48.4%	0.545			
Others (well, moderately)	119 (86%)	69.2%	61.1%				
**IM**							
**(+)**	19 (14%)	34.1%	22.7%	**< 0.001**	3.675	1.848–7.308	**< 0.001**
**(−)**	120 (86%)	77.1%	66.0%				
Vp							
(+)	26 (19%)	49.6%	42.5%	**0.009**	1.700	0.940–3.649	0.130
(−)	113 (81%)	74.6%	62.8%				
**Low SMI**	86 (62%)	63.6%	52.4%	**0.039**	2.006	1.012–3.974	**0.046**
**High SMI**	53 (38%)	80.9%	70.5%				
Blood transfusion							
(+)	22 (16%)	40.0%	30.0%	**0.002**	2.012	1.001–4.045	0.050
(−)	117 (84%)	75.5%	64.3%				

Variables in bold are statistically significant (*P* < 0.05)

*Abbreviations: BMI* Body mass index, *ALBI* albumin-bilirubin, *HBV* hepatitis type B, *HCV* hepatitis type C, *DM* diabetes mellitus, *NLR* neutrophil-to-lymphocyte ratio, *PLT* platelets, *Hb* hemoglobin, *PT* prothrombin time, *AST* aspartate aminotransferase, *ALT* alanine aminotransferase, *CHE* cholinesterase, *Alb* albumin, *T-chol* total-cholesterol, *PIVKAII* protein induced by vitamin K absence or antagonist-II, *AFP* α-fetoprotein, *IM* intrahepatic metastasis, *VP* portal vain invasion, *SMI* skeletal muscle mass index

**Table 2:** Prognostic factors for recurrence-free survival identified by univariate and multivariate analyses (n = 139)

				Univariate	Multivariate		
Variables	n (%)	3-year survival	5-year survival	*P*-value	HR	95% CI	*P*-value
Male sex	110 (79%)	45.8%	41.2%	0.180			
Female sex	29 (21%)	56.6%	40.4%				
Age (years)							
≤ 80	109 (78%)	24.9%	24.9%	0.084			
> 80	30 (22%)	52.4%	45.2%				
BMI (kg/m^2^)							
> 25	34 (25%)	48.0%	48.0%	0.699			
≤ 25	105 (75%)	48.3%	39.3%				
ALBI							
grade III	30 (22%)	49.5%	44.6%	0.708			
< grade III	109 (78%)	47.7%	40.1%				
HBV							
(+)	27 (19%)	63.2%	47.7%	0.236			
(−)	112 (81%)	44.1%	37.7%				
HCV							
(+)	70 (50%)	40.8%	38.6%	0.163			
(−)	69 (50%)	56.4%	44.2%				
DM							
(+)	29 (21%)	57.5%	43.6%	0.674			
(−)	110 (79%)	45.2%	39.9%				
NLR							
≥ 4	13 (9%)	19.4%	19.4%	0.279			
< 4	126 (91%)	49.9%	42.5%				
PLT (×10^4^/μL)							
normal (13–35)	97 (70%)	50.4%	42.4%	0.595			
abnormal	42 (30%)	44.1%	39.2%				
Hb (g/dL)							
normal (13.5–15.8)	95 (68%)	50.5%	41.6%	0.795			
abnormal	44 (32%)	44.3%	40.9%				
PT (%)							
normal (70–130)	125 (90%)	50.5%	42.5%	0.125			
abnormal	14 (10%)	28.8%	28.8%				
AST (U/L)							
normal (13–33)	71 (51%)	45.6%	33.9%	0.888			
abnormal	68 (49%)	51.1%	48.1%				
ALT (U/L)							
normal (8–42)	100 (72%)	48.8%	41.0%	0.770			
abnormal	39 (28%)	46.8%	40.9%				
CHE (g/dL)							
normal (229–521)	77 (55%)	53.8%	44.9%	0.251			
abnormal	62 (45%)	41.7%	36.4%				
Alb (g/dL)							
normal (4.0–5.0)	115 (83%)	48.5%	39.8%	0.878			
abnormal	24 (17%)	46.7%	46.7%				
T-chol (mg/dL)							
normal (128–219)	118 (85%)	49.4%	43.0%	0.907			
abnormal	20 (14%)	42.1%	31.6%				
**PIVKA-II (mAU/mL)**							
**normal (< 40)**	62 (45%)	61.0%	51.0%	**0.048**	1.321	0.778–2.240	0.320
**abnormal**	77 (55%)	37.7%	33.0%				
**AFP (ng/mL)**							
**normal (> 10)**	68 (49%)	58.8%	48.9%	**0.036**	1.612	0.960–2.707	0.071
**abnormal**	68 (49%)	38.7%	33.5%				
**Tumor number**							
**solitary**	113 (81%)	51.8%	44.5%	**0.025**	1.810	1.025–3.197	**0.041**
**multiple**	26 (19%)	31.4%	18.8%				
Tumor size							
3 cm <	71 (51%)	49.5%	43.4%	0.938			
≤ 3 cm	54 (39%)	52.6%	42.7%				
Poor differentiation	20 (14%)	42.1%	28.1%	0.337			
Others (well, moderately)	119 (86%)	49.3%	43.6%				
**IM**							
**(+)**	19 (14%)	5.6%	5.6%	**< 0.001**	4.115	2.255–7.510	**< 0.001**
**(−)**	120 (86%)	56.1%	47.5%				
**Vp**							
**(+)**	26 (19%)	28.4%	28.4%	**0.016**	1.490	0.824–2.695	0.187
**(−)**	113 (81%)	52.8%	43.5%				
Low SMI	86 (62%)	46.1%	39.8%	0.335			
High SMI	53 (38%)	51.9%	43.9%				
**Blood transfusion**							
**(+)**	22 (16%)	24.6%	24.6%	**0.008**	2.288	1.244–4.207	**0.008**
**(−)**	117 (84%)	52.4%	44.4%				

Variables in bold are statistically significant (*P* < 0.05). Abbreviations: BMI, Body mass index; ALBI, albumin-bilirubin; HBV, hepatitis type B; HCV, hepatitis type C; DM, diabetes mellitus; NLR, neutrophil-to-lymphocyte ratio; PLT, platelets; Hb, hemoglobin; PT, prothrombin time; AST, aspartate aminotransferase; ALT, alanine aminotransferase; CHE, cholinesterase; Alb, albumin; T-chol, total-cholesterol; PIVKAII, protein induced by vitamin K absence or antagonist-II; AFP, α-fetoprotein; IM, intrahepatic metastasis; Vp, portal vain invasion; SMI, skeletal muscle mass index

### Characteristics of low SMI patients and high SMI patients with HCC

Supplemental Table 1 provides a comparison of the perioperative characteristics between low SMI and high SMI patients with HCC. The male ratio in the low SMI group was higher than that in the high SMI group. The number of patients with low body mass index was significantly higher in the low SMI group than that in high SMI group. No significant differences were observed for age, NLR, PNI, and GPS between the groups. Tumor markers, liver function, and tumor-related factors were compatible between the two groups. The pattern of recurrence and type of treatment were not different between the two groups.

### Characteristics of patients with HCC who received blood transfusion and those who did not receive blood transfusion

Table 3 presents the perioperative characteristics of the blood transfusion group and non-transfusion group with HCC. PNI of the transfusion group was lower than that of the non-transfusion group. With regard to the laboratory data, albumin levels and PIVKA-II were significantly higher in the transfusion group than those in the non-transfusion group. The preoperative hemoglobin level was significantly lower in the patients who received blood transfusion than in those who did not. No significant differences were observed in the tumor-related factors between the two groups. Operation time and recurrence ratio were significantly higher in the transfusion group than those in the non-transfusion group. Even after the propensity-matched analysis, blood transfusion showed a negative impact on RFS.

**Table 3:** Comparison of the characteristics of patients who underwent transfusion and those who did not undergo transfusion with data reported for the whole study series and for one-to-one score-matched pair

	Whole study series					Propensity score-matched series		
	All patients (*n* = 139)	Transfusion (*n* = 22)	Non-transfusion (*n* = 117)	*P*-value	All patients (*n* = 40)	Transfusion (*n* = 20)	Non-transfusion (n = 20)	*P*-value
Male sex	110 (79%)	18 (82%)	92 (79%)	1.000	31 (78%)	16 (80%)	15 (75%)	1.000
Age (years) ≥70	88 (63%)	16 (73%)	72 (62%)	0.348	27 (68%)	15 (75%)	12 (60%)	0.501
BMI (kg/m^2^) ≥25.0	34 (25%)	4 (18%)	30 (26%)	0.593	10 (25%)	4 (20%)	6 (30%)	0.716
HBV	27 (19%)	3 (14%)	24 (21%)	0.568	5 (13%)	3 (15%)	2 (10%)	1.000
HCV	70 (50%)	12 (55%)	58 (50%)	0.817	20 (50%)	10 (50%)	10 (50%)	1.000
DM	29 (21%)	2 (9%)	27 (23%)	0.165	7 (18%)	2 (10%)	5 (25%)	0.407
NLR	13 (9%)	1 (5%)	12 (10%)	0.481	4 (10%)	1 (5%)	2 (10%)	1.000
**PNI**	11 (8%)	**5 (23%)**	**6 (5%)**	**0.015**	7 (18%)	5 (25%)	2 (10%)	0.407
GPS ≥1	24 (17%)	7 (32%)	17 (15%)	0.065	13 (33%)	6 (30%)	7 (35%)	1.000
**Hb (g/dL)**	14.1 (9.4–18.2)	**13.5 (9.4–17.8)**	**14.1 (9.4–18.2)**	**0.016**	13.4 (9.4–17.8)	13.7 (9.4–17.8)	13.2 (9.4–15.3)	0.490
PLT (×10^4^/μL) < 14	45 (32%)	8 (36%)	37 (32%)	0.804	13 (33%)	7 (35%)	6 (30%)	1.000
PT (%) < 70	14 (10%)	1 (5%)	13 (11%)	0.698	2 (5%)	1 (5%)	1 (5%)	1.000
T-Bil (mg/dL) **≥1**	29 (21%)	4 (14%)	25 (21%)	0.495	6 (15%)	3 (15%)	3 (15%)	1.000
AST (U/L) ≥ 38	57 (41%)	11 (50%)	46 (39%)	0.357	19 (48%)	11 (55%)	8 (40%)	0.527
ALT (U/L) ≥42	41 (30%)	7 (32%)	34 (29%)	0.802	16 (40%)	7 (35%)	9 (45%)	0.748
ChE (g/dL) < 100	3 (2%)	1 (5%)	2 (2%)	0.406	2 (5%)	1 (5%)	1 (5%)	1.000
Alb (g/dL) < 4	63 (45%)	12 (55%)	51 (44%)	0.361	26 (65%)	10 (50%)	16 (80%)	0.096
ICGR15 (%) **≥ 10**	93 (67%)	18 (82%)	75 (64%)	0.059	33 (83%)	18 (90%)	15 (75%)	0.407
AFP (ng/mL) ≥ 10	68 (49%)	15 (68%)	53 (45%)	0.102	24 (60%)	13 (65%)	11 (55%)	0.748
**PIVKA-II (mAU/mL) ≥ 40**	96 (69%)	**21 (96%)**	**75 (64%)**	**0.004**	35 (88%)	19 (95%)	16 (80%)	0.342
Tumor diameter > 2 cm	112 (81%)	20 (91%)	92 (79%)	0.249	37 (93%)	18 (90%)	19 (95%)	1.000
Tumor number	1 (1–5)	1 (1–5)	1 (1–3)	0.963	1 (1–3)	1 (1–3)	1 (1–3)	0.495
Poor differentiation	20 (14%)	3 (14%)	17 (15%)	1.000	4 (10%)	3 (15%)	1 (5%)	0.605
IM (+)	19 (6%)	4 (18%)	15 (13%)	0.738	4 (10%)	2 (10%)	2 (10%)	1.000
**Operation time (min)**	303 (66–591)	**383 (210–591)**	**282 (66–582)**	**< 0.001**	374 (127–591)	383 (210–591)	348 (127–582)	0.461
**Intraoperative blood loss (g)**	506 (0–6055)	**820 (250–6055)**	**230 (0–2467)**	**< 0.001**	500 (40–6055)	**820 (250–6055)**	**450 (40–1181)**	**0.002**
Hospital stay (days)	22 (5–100)	20 (11–80)	17 (5–100)	0.058	20 (11–81)	20 (11–88)	20 (15–81)	0.820

Variables in bold are statistically significant (*P*<0.05). Continuous variables are expressed as medians (range). Qualitative variables are expressed as numbers (%). Abbreviations: BMI, Hb; hemoglobin, Body mass index; HBV, hepatitis type B; HCV, hepatitis type C, DM, diabetes mellitus; NLR, neutrophil-to-lymphocyte ratio; PNI, prognostic nutritional index; GPS, Glasgow prognostic score; PLT, platelets; PT, prothrombin time; T-Bil, total-bilirubin; AST, aspartate aminotransferase; ALT, alanine aminotransferase; ChE, cholinesterase; Alb, albumin; ICGR15, indocyanine green retention15; AFP, α-fetoprotein; PIVKA-II, protein induced by vitamin K absence or antagonist-II; IM, intrahepatic metastasis

## Discussion

Several studies examining the prognosis of HCC patients following surgery have traditionally emphasized the effects of tumor-specific variables, lymph node metastasis, IM, and vascular invasion [19–22]. Undoubtedly, tumor-specific factors were related to the long-term prognosis; however, patient-related factors such as the immunological variables and sarcopenia have been reported as significant factors affecting the long-term prognosis. In the present study, low SMI and perioperative blood transfusion had a strong impact on long-term prognosis. Preoperative detection of low SMI is important to assess the prognosis in patients with HCC after curative surgery. Frailty is widely used as a metric of patient physiological reserve and overall health status. Recent studies have shown that skeletal muscle mass, which can be measured on CT cross-sectional imaging, is a marker of frailty and is used to detect sarcopenia [23, 24]. On the other hand, the European Working Group on Sarcopenia in Older People recommended that sarcopenia should be diagnosed if both low muscles and low muscle function are present [25]. The efficacy of preoperative exercise and nutrition in patients with sarcopenia remains unclear; several studies have demonstrated that aerobic and resistance exercises are more effective in improving upper lower body muscle strength than the usual treatment [26, 27]. In addition, the skeletal muscle was recently recognized as an endocrine organ [28]. Interleukin (IL)-6, which may influence liver metabolism, is released from the skeletal muscle. IL-6 has already been identified as a factor with biological effects in patients with liver fibrosis and HCC [28]. Insulin-like growth factor (IGF)-1 was confirmed as a stimulatory factor in the development and regulation of skeletal muscle mass [28]. IGF-1 is mainly produced by the liver. Therefore, serum IGF-1 levels were low in patients with sarcopenia, and low IGF-1 levels result in the progression of HCC. Hence, there is a relationship between sarcopenia and HCC prognosis. Preventing muscle wasting is important for improving the prognosis of patients with HCC. In particular, patients with liver cirrhosis have decreased liver function, glycogen stores, and protein synthesis due to liver atrophy. In our study, we found no significant difference between the patients with low and high SMIs. Regardless of the equivalent recurrence rates, the patients with low SMI showed poorer OS than those with high SMI. This is affected by the treatment strategy for HCC recurrence; that is, the patients with low SMI did not undergo a second hepatectomy unlike those with high SMI. Their consumption of amino acids from the skeletal muscle as an energy source increases, which causes progression of sarcopenia [29, 30]. There is a report showing that perioperative nutritional therapy using branched-chain amino acids improves OS of patients with cirrhosis and sarcopenia [31]. Multidisciplinary approach to overcome sarcopenia would improve the long-term prognosis of patients with HCC following curative surgery.

Several previous studies have demonstrated that blood transfusion had a negative impact on the prognosis of HCC patients [6, 32]. In line with Harada et al.’s study, this study suggests that blood transfusion was associated with HCC recurrence after hepatectomy in patients with Child–Pugh class A. Recent studies have reported that transfusion-related immunomodulation affects the prognosis of patients who received blood transfusion. RBCs transfusion was referred to as an immune system suppressor and has been linked to tumor recurrence [33]. The absolute peripheral blood lymphocyte count of patients who underwent blood transfusion is lower than that of patients who did not undergo blood transfusion [34]. There is one study that demonstrated that the natural killer cell activity of patients who underwent blood transfusion decreased on postoperative day 7 [35], leading to decreased tumor suppression. Additionally, blood transfusion cause secondary iron overload, which may accelerate the progression of liver fibrosis and recurrence of HCC [36]. The long-term prognosis of patients with distal cholangiocarcinoma who received perioperative RBCs transfusion was poorer than those who did not receive RBCs transfusion [37]. Moreover, intraoperative RBCs transfusion was associated with poor OS in patients with periampullary cancer who underwent pancreaticoduodenectomy [38]. In our study, the patients in the blood transfusion group required longer operation time and had a larger volume of intraoperative blood loss than those in the non-transfusion group even after the propensity-matched analysis. Blood transfusion had a negative impact on RFS. Hence, it is important to avoid unnecessary blood transfusion and intraoperative blood loss to maintain the normal function of the host immune system.

This retrospective, single-center study had a limited sample size. Future prospective cohort studies involving multiple institutions should be performed to confirm our results. The patients who underwent blood transfusion were significantly associated with preoperative anemia as compared with those who did not receive blood transfusion. The several biases were still attributed to the enforcement of blood transfusion even after the propensity-matched analysis.

## Conclusion

In conclusion, low SMI and perioperative blood transfusion were associated with long-term prognosis of HCC patients with Child–Pugh class A after curative surgery. Low SMI as an indicator of nutritional status was considered as an independent prognostic factor in patients with HCC. Transfusion-related immune response was also strongly affected by recurrence rates in patients with HCC.

## Supplementary information

**Additional file 1: Supplemental Fig. 1a, b.** Kaplan-Meier curves for 5-year recurrence-free survival rates in propensity score-matched hepatocellular carcinoma patients stratified according to transfusion. Transfusion patients are represented by the thick solid line, and non-transfusion patients are represented by the thin solid line.

**Additional file 2: Supplemental Table 1.** Characteristics of low SMI and high SMI patients with hepatocellular carcinoma.

## Data Availability

The datasets used and/or analyzed during the current study available from the corresponding author on reasonable request.

## Abbreviations

SMI: skeletal muscle mass index
HCC: hepatocellular carcinoma
OS: overall survival
RFS: recurrence-free survival
IM: intrahepatic metastasis
HBV: hepatitis B
DFS: disease-free survival
GPS: Glasgow Prognostic Score
NLR: neutrophil-to-lymphocyte ratio
RBCs: red blood cells
HU: Hounsfield unit
CT: computed tomography
PNI: prognostic nutritional index
PIVKA-II: protein induced by vitamin K absence or antagonist-II
AFP: α-fetoprotein
IL: interleukin
IGF: insulin-like growth factor
