# Supplementary figures and images for: Impact of low skeletal muscle mass index and perioperative blood transfusion on the prognosis for HCC following curative resection
Source: BMC Gastroenterol. 2020 Oct 7;20:328. doi: 10.1186/s12876-020-01472-z (PMC7539410; doi:10.1186/s12876-020-01472-z)

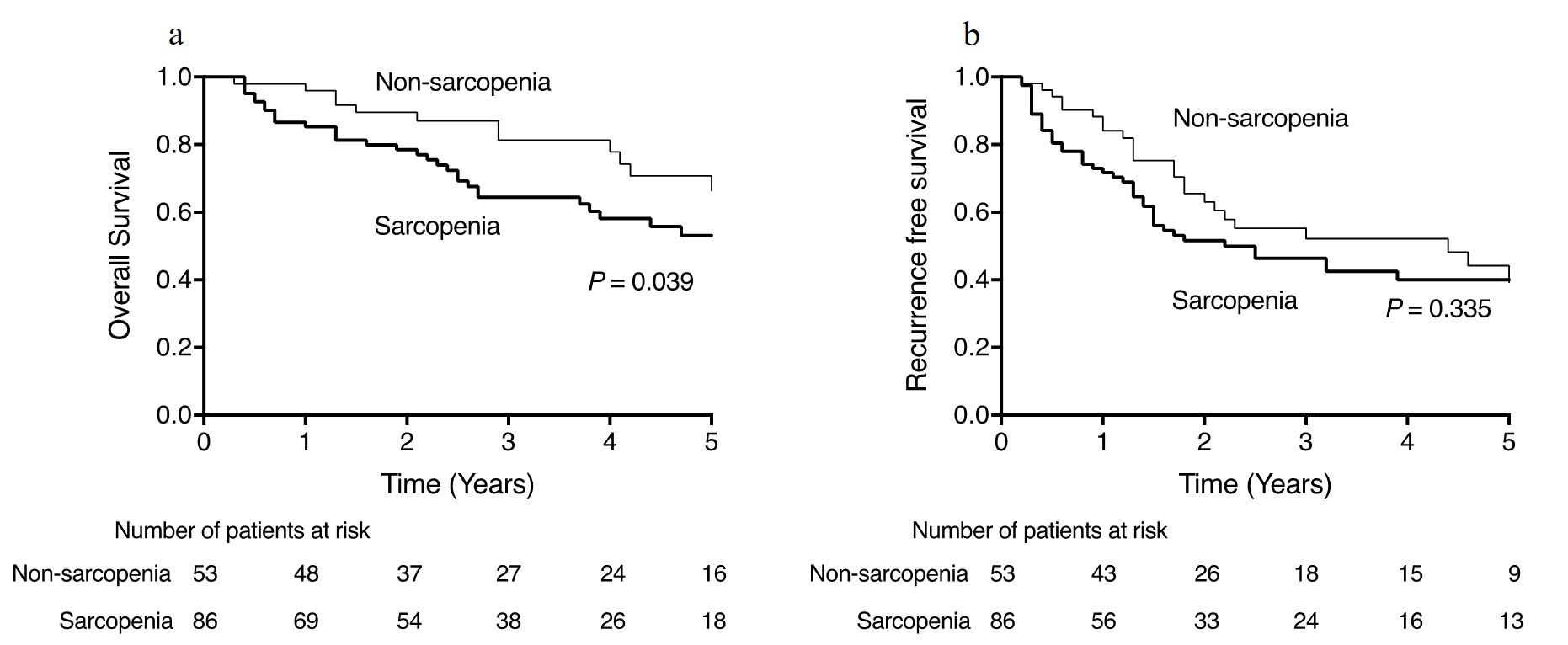

Supplement: Supplementary file 1 — Additional file 1: Supplemental Fig. 1a, b. Kaplan-Meier curves for 5-year recurrence-free survival rates in propensity score-matched hepatocellular carcinoma patients stratified according to transfusion. Transfusion patients are represented by the thick solid line, and non-transfusion patients are represented by the thin solid line. [file 12876_2020_1472_MOESM1_ESM.jpg]
