# Supplementary material for: Impact of low skeletal muscle mass index and perioperative blood transfusion on the prognosis for HCC following curative resection
Source: BMC Gastroenterol. 2020 Oct 7;20:328. doi: 10.1186/s12876-020-01472-z (PMC7539410; doi:10.1186/s12876-020-01472-z)
Supplement: Supplementary file 2 — Additional file 2: Supplemental Table 1. Characteristics of low SMI and high SMI patients with hepatocellular carcinoma. [file 12876_2020_1472_MOESM2_ESM.docx]

Supplemental table 1. Characteristics of low SMI and high SMI patients with hepatocellular carcinoma

|  | All patients  (n=139) | Low SMI patients  (n=86) | High SMI patients  (n=53) | *P*-value |
| --- | --- | --- | --- | --- |
| **Male sex** | 110 (79%) | 80 (93%) | 30 (57%) | **<0.001** |
| Age (years) | 72 (32-92) | 73 (36-92) | 72 (32-84) | 0.155 |
| **BMI (kg/m^2^)** | 23 (16-33) | 21(16-27) | 25 (18-33) | **<0.001** |
| HBV | 27 (19%) | 16 (19%) | 11 (21%) | 0.826 |
| HCV | 70 (50%) | 42 (49%) | 28 (53%) | 0.728 |
| DM | 29 (21%) | 17 (20%) | 12 (23%) | 0.830 |
| NLR | 2 (0.3-7.4) | 2 (0.3-7.4) | 2 (0.5-6.1) | 1.000 |
| **PNI** | 47.4 (34.3-59.9) | 47.1 (36.3-59.9) | 47.9 (34.3-58.8) | 0.272 |
| GPS ≥1 | 24 (17%) | 17 (20%) | 7 (13%) | 0.386 |
| PLT (×10^4^/μL) | 19 (4-51) | 17 (4-51) | 19 (6-49) | 0.376 |
| PT (%) | 84 (56-128) | 83 (56-109) | 84 (59-128) | 0.887 |
| T-Bil (mg/dL) | 0.8 (0.2-2.0) | 0.7 (0.2-1.8) | 0.7 (0.3-2.0) | 0.929 |
| AST (U/L) | 42 (11-130) | 35 (14-127) | 29 (11-130 | 0.245 |
| ALT (U/L) | 37 (7-173) | 31 (7-173) | 28 (7-117) | 0.189 |
| ChE (g/dL) | 234 (37-412) | 231 (86-410) | 242 (37-412) | 0.143 |
| Alb (g/dL) | 4 (3-5) | 4 (3-5) | 4 (3-5) | 0.686 |
| ICGR15 (%) | 13 (0.1-89) | 12 (0.1-89) | 12 (0.1-75) | 1.000 |
| AFP (ng/mL) | 10 (2-223330) | 9 (2-41013) | 12 (2-233330) | 0.859 |
| PIVKA-II (mAU/mL) | 142 (12-675000) | 147 (12-675000) | 129 (14-39000) | 0.726 |
| Tumor diameter | 34 (5-150) | 38 (5-150) | 30 (13-150) | 0.149 |
| Tumor number | 1 (1-5) | 1 (1-5) | 1 (1-3) | 0.655 |
| Poor differentiation | 20 (14%) | 13 (15%) | 7 (13%) | 0.809 |
| IM (+) | 19 (6%) | 13 (15%) | 6 (11%) | 0.617 |
| Operation time (min) | 303 (66-591) | 306 (89-591) | 299 (66-582) | 0.732 |
| Intraoperative blood loss (g) | 506 (0-6055) | 569 (0-6055) | 405 (0-2100) | 0.551 |
| Blood transfusion | 22 (16%) | 18 (21%) | 4 (8%) | 0.085 |
| Hospital stay (days) | 22 (5-100) | 21 (5-81) | 24 (11-100) | 0.920 |
| Recurrence | 74 (53%) | 46 (53%) | 28 (53%) | 1.000 |

Variables in bold are statistically significant (*P*＜0.05). Continuous variables are expressed as medians (range). Qualitative variables are expressed as numbers (%). Abbreviations: SMI, skeletal muscle mass index; BMI, Body mass index; HBV, hepatitis type B; HCV, hepatitis type C; DM, diabetes mellitus; NLR, neutrophil-to-lymphocyte ratio; PNI, prognostic nutritional index; GPS, Glasgow prognostic score; PLT, platelets; PT, prothrombin time; T-Bil, total-bilirubin; AST, aspartate aminotransferase; ALT, alanine aminotransferase; ChE, cholinesterase; Alb, albumin; ICGR15, indocyanine green retention15; AFP, α-fetoprotein; PIVKA-II, protein induced by vitamin K absence or antagonist-II; IM, intrahepatic metastasis
